# Supplementary material for: Associations of Glucometabolic Indices with Aortic Stiffness in Patients Undergoing Peritoneal Dialysis with and without Diabetes Mellitus
Source: Int J Mol Sci. 2023 Dec 4;24(23):17094. doi: 10.3390/ijms242317094 (PMC10707165; doi:10.3390/ijms242317094)
Supplement: Supplementary file 1 [file ijms-24-17094-s001.zip › ijms-2710742-supplementary.pdf]

Table S1. Associations of glycemic parameters with aortic PWV among non-DM patients undergoing PD, further stratified by gender.

Variables	Aortic PWV (m/s)							
	Male (n=46)				Female (n=64)			
	Unadjusted		Adjusted		Unadjusted		Adjusted	
	β (95% CI)	*P*	β (95% CI)	*P*	β (95% CI)	*P*	β (95% CI)	*P*
**Fasting glucose**								
Normal (<100 mg/dl)	Reference	0.115	Reference	0.094	Reference	0.005*	Reference	0.097
Impaired (≥ 100mg/dl)	0.66 (-0.17, 1.48)		0.78 (-0.14, 1.71)		0.87 (0.27, 1.48)		0.60 (-0.11, 1.32)	
**HOMA-IR status**								
Low (≤ 4.91)	Reference	0.005*	Reference	0.002*	Reference	0.349	Reference	0.535
High (> 4.91)	1.14 (0.36, 1.91)		1.65 (0.66, 2.65)		0.30 (-0.34, 0.94)		0.20 (-0.44, 0.83)	
**Serum AGEs level**[¶]								
Low (≤ 2.5 ng/ml)	Reference	0.064	Reference	0.228	Reference	0.258	Reference	0.323
High (>2.5 ng/ml)	0.80 (-0.05, 1.65)		0.69 (-0.45, 1.83)		-0.37 (-1.02, 0.28)		-0.33 (-0.99, 0.33)	
**PD glucose load**								
Low (≤ 170 g/day)	Reference	0.460	Reference	0.328	Reference	0.138	Reference	0.170
High (>170 g/day)	0.34 (-0.58, 1.25)		0.50 (-0.53, 1.54)		0.50 (-0.16, 1.15)		0.45 (-0.20, 1.09)	
**Icodextrin**								
Non-user	Reference	0.855	Reference	0.987	Reference	0.866	Reference	0.777
User	-0.08 (-0.96, 0.80)		-0.01 (-1.04, 1.05)		0.07 (-0.70, 0.82)		0.10 (-0.60, 0.80)	

The adjusted models adopted age, log-PD duration, hypertension, hyperlipidemia, BMI, WC, systolic BP, Ca × P, total Kt/V, total cholesterol, Hb, albumin, and statins as covariates.

[¶]Serum AGEs levels were not measured four patients, two in male group and two in female group.

Table S2. Associations of glycemic parameters with aortic PWV among DM patients undergoing PD, further stratified by gender.

Variables	Aortic PWV (m/s)							
	Male (n=31)				Female (n=31)			
	Unadjusted		Adjusted		Unadjusted		Adjusted	
	**β (95% CI)**	***P***	**β (95% CI)**	***P***	**β (95% CI)**	***P***	**β (95% CI)**	***P***
**HbA1c (%)**								
≤ 7%	Reference	0.131	Reference	0.125	Reference	0.927	Reference	0.470
> 7%	-1.17 (-2.71, 0.37)		-1.72 (-3.96, 0.53)		-0.05 (-1.20, 1.10)		0.50 (-0.94, 1.94)	
**HOMA-IR status**								
Low (≤ 4.91)	Reference	0.795	Reference	0.388	Reference	0.461	Reference	0.844
High (> 4.91)	-0.20 (-1.73, 1.34)		1.04 (-1.44, 3.52)		-0.40 (-1.50, 0.70)		0.11 (-1.05, 1.27)	
**Serum AGEs level**[¶]								
Low (≤ 2.5 ng/ml)	Reference	0.974	Reference	0.970	Reference	0.819	Reference	0.461
High (>2.5 ng/ml)	-0.03 (-1.61, 1.56)		-0.04 (-2.26, 2.18)		0.13 (-1.02, 1.28)		-0.52 (-1.98, 0.94)	
**PD glucose load**								
Low (≤ 170 g/day)	Reference	0.722	Reference	0.594	Reference	0.806	Reference	0.571
High (>170 g/day)	0.29 (-1.35, 1.92)		0.81 (-2.34, 3.95)		0.14 (-1.04, 1.33)		-0.43 (-2.01, 1.15)	
**Icodextrin**								
Non-user	Reference	0.512	Reference	0.659	Reference	0.781	Reference	0.559
User	0.84 (-1.74, 3.41)		0.76 (-2.83, 4.35)		0.21 (-1.30, 1.71)		-0.60 (-2.75, 1.55)	

The adjusted models adopted age, log-PD duration, hypertension, hyperlipidemia, BMI, WC, systolic BP, Ca × P, total Kt/V, total cholesterol, Hb, albumin, and statins as covariates.

[¶]Serum AGEs levels were not measured two patients, one in male group and one in female group.
